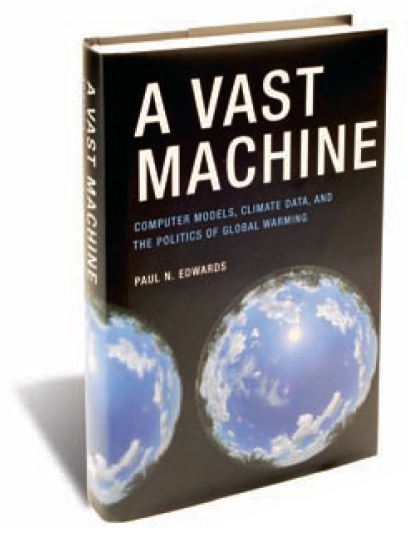# A Vast Machine: Computer Models, Climate Data, and the Politics of Global Warming

**Published:** 2011-04

**Authors:** Robert S. Chen

**Affiliations:** Robert S. Chen directs CIESIN, an interdisciplinary research center in Columbia University’s Earth Institute. A geographer, he manages the NASA Socioeconomic Data and Applications Center, co-leads the IPCC Data Distribution Centre, and is active in international data sharing and preservation initiatives. He staffed many early National Research Council climate change reports

In *A Vast Machine,* Paul Edwards documents the evolution of a broad scientific field that began with the curiosity of a few 19th-century explorers and scholars and now spans a worldwide community of scientists, engineers, and other specialists working with huge quantities of data, immensely complex computer models, and many sophisticated instruments and measurement platforms. As a scholar in science and technology studies, Edwards provides historical context and insightful perspectives on how observations of Earth’s weather and climate have shaped scientific theories and models underpinning weather prediction and climate change—and how these in turn have dramatically affected our “knowledge infrastructure”: the ways in which environmental measurements are now made, analyzed, interpreted, and used both in science and in global debates about environmental policy. In particular, he elucidates what scientists often take for granted—that models and observational data *together* form an inseparable basis for scientific understanding and prediction—in the context of current policy debates that have often tried to characterize observational data as independent, immutable representations of “truth” and computer models as imperfect tools subject to scientific bias and error.

A compelling aspect of *A Vast Machine* is Edwards’ careful history of the development of weather observations and prediction in conjunction with the simultaneous and often intertwined evolution of climate monitoring and modeling. Weather data networks and numerical weather prediction models have grown rapidly in response to immediate societal needs and interests, evolving into ubiquitous technologies and well-oiled systems for delivering useful information to a wide range of users. Climate data networks and climate models share many similar components and approaches, but require a shift in perspective in analyzing and using data and model predictions. For example, long time series of climate data need continual reassessment and reanalysis because improvements in our current knowledge and understanding of what determines and influences climate also affect how we interpret measurements made in the past.

Because the global climate system is so complex and interconnected across land, oceans, atmosphere, the cryosphere, and the biosphere, the process of making observations collected from around the world—even through Earth-orbiting satellites—into truly “global” data sets requires significant innovation and investment. Edwards identifies different types of “data friction” that affect the flows and transformations of data and information, not only technical bottlenecks and constraints but also challenges stemming from coordinating scientists and systems across different disciplines, countries, and cultures. He also examines the institutional and structural histories of national agencies, international organizations, and other stakeholders that have shaped the evolution of weather and climate science and observational systems, including the strong influence of war, the military, politics, and the emergence of global environmental institutions and awareness.

For anyone interested in global warming or more generally in climate or weather issues, *A Vast Machine* is well worth a careful read. It provides an unusually broad and long-term view of the development of climate science and associated climate data, models, and information infrastructure, supplemented by useful figures and very detailed notes and references. Edwards begins with helpful guidance on what chapters might be of strongest interest to some readers or too technical for others. For those more generally interested in science, science and technology policy, and data and information management issues, he offers a sprinkling of comparisons with analogous issues and pointers to relevant literature.

As someone who has been closely involved for several decades in many of the research, data, and assessment activities and institutions documented in this volume, I found it indeed a rare and unexpected opportunity to learn so much about how these endeavors have fit into a vast and vibrant scientific enterprise that is of critical importance to the sustainability of our planet. The volume is also a testament to the vision and breadth of interests of the late Stephen Schneider of Stanford University, who helped Edwards with many aspects of his research (and who was a key mentor for this reviewer as well).

## Figures and Tables

**Figure:**